# A Review of the Influence of Various Extraction Techniques and the Biological Effects of the Xanthones from Mangosteen (*Garcinia mangostana* L.) Pericarps

**DOI:** 10.3390/molecules27248775

**Published:** 2022-12-10

**Authors:** Vasin Yuvanatemiya, Pao Srean, Wiyada Kwanhian Klangbud, Karthikeyan Venkatachalam, Jittimon Wongsa, Thanya Parametthanuwat, Narin Charoenphun

**Affiliations:** 1 Faculty of Marine Technology, Burapha University, Chanthaburi Campus, Thamai, Chanthaburi 22170, Thailand; 2 Faculty of Agriculture and Food Processing, National University of Battambang, Battambang 020101, Cambodia; 3 Center of Excellence Research for Melioidosis and Microorganisms, School of Allied Health Sciences, Walailak University, Thasala, Nakhon Si Thammarat 80160, Thailand; 4 Faculty of Innovative Agriculture and Fishery Establishment Project, Prince of Songkla University, Surat Thani Campus, Makham Tia, Mueang, Surat Thani 84000, Thailand; 5 Department of Agricultural Engineering for Industry, Faculty of Industrial Technology and Management, King Mongkut’s University of Technology North Bangkok, Prachinburi Campus, Prachinburi 25230, Thailand; 6 Faculty of Science and Arts, Burapha University, Chanthaburi Campus, Thamai, Chanthaburi 22170, Thailand

**Keywords:** extraction, process, mangosteen, xanthones, pericarps, by-products

## Abstract

Xanthones are significant bioactive compounds and secondary metabolites in mangosteen pericarps. A xanthone is a phenolic compound and versatile scaffold that consists of a tricyclic xanthene-9-one structure. A xanthone may exist in glycosides, aglycones, monomers or polymers. It is well known that xanthones possess a multitude of beneficial properties, including antioxidant activity, anti-inflammatory activity, and antimicrobial properties. Additionally, xanthones can be used as raw material and/or an ingredient in many food, pharmaceutical, and cosmetic applications. Although xanthones can be used in various therapeutic and functional applications, their properties and stability are determined by their extraction procedures. Extracting high-quality xanthones from mangosteen with effective therapeutic effects could be challenging if the extraction method is insufficient. Although several extraction processes are in use today, their efficiency has not yet been rigorously evaluated. Therefore, selecting an appropriate extraction procedure is imperative to recover substantial yields of xanthones with enhanced functionality from mangosteens. Hence, the present review will assist in establishing a precise scenario for finding the most appropriate extraction method for xanthones from mangosteen pericarp by critically analyzing various conventional and unconventional extraction methods and their ability to preserve the stability and biological effects of xanthones.

## 1. Introduction

Mangosteen (*Garcinia mangostana* L.) is one of the essential commercial fruits of Thailand and also in many countries in Southeast Asia and is generally recognized as the queen of fruit as its appearance has petals on the head that resembles a queen’s crown. It is a round fruit that is green when unripe and turns dark purple upon fully ripe. The fruit contains several petals of white, consumable flesh, which has a sweet taste, and the number of petals can be easily known by the calyxes present beneath the fruits. Thailand is the second largest producer after Indonesia in Southeast Asia, whereas India is the largest producer in the world. From Thailand, the export of mangosteen in 2020 was 292,147 t accounting for 409,466 USD. The export was divided into two main categories, including fresh mangosteen (291,951 t) and frozen mangosteen (196 t) [1]. The demand for mangosteen in the world market will likely increase and expand continuously if the product has been certified with good agricultural practice standards. The main markets for fresh mangosteen from Thailand are China, Vietnam, and Hong Kong, while frozen mangosteen is exported to South Korea, Taiwan, and the United States [2]. Transporting fresh mangosteen is highly problematic due to the transportation delay and cost; compared with the costlier air transport, ocean transport is inexpensive, and, due to time consumption, the fruits get spoiled before they reach their destination, which restricts the expansion of the distribution chain of fresh mangosteen fruits. Therefore, the transportation of fresh mangosteen is a significant problem for exporters. Exporting processed mangosteen products is a promising means to resolve the transportation problem of fresh mangosteen and increase the product’s shelf life. There are several types of processed products of mangosteen, such as frozen mangosteen, mangosteen juice, mangosteen jam, stirred mangosteen, mangosteen cider, and mangosteen vinegar [3].

**Figure 1:**
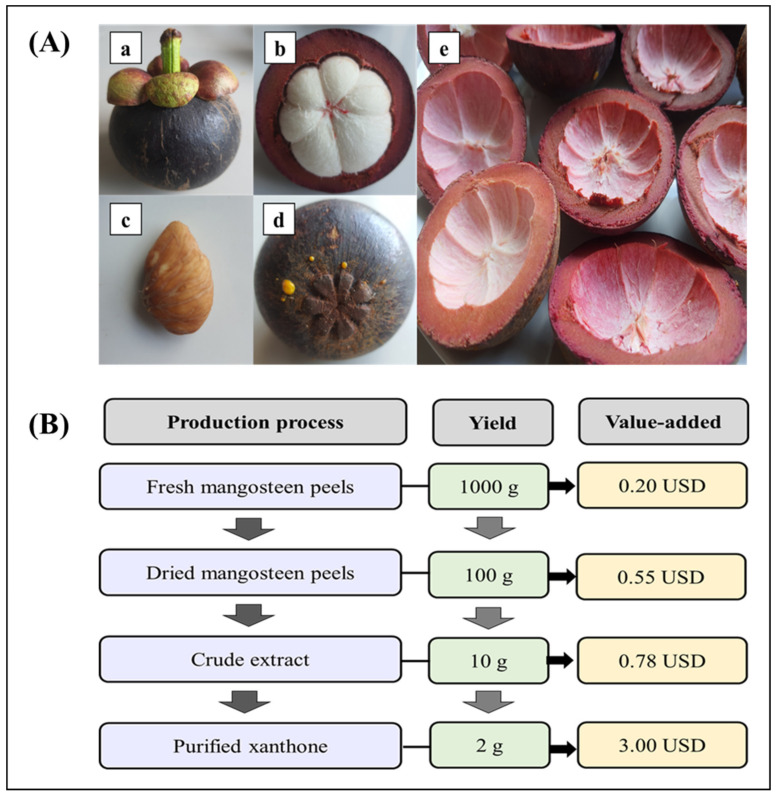
Mangosteen fruit and its parts ((**a**) fruit, (**b**) pulp, (**c**) seed, (**d**) sepals under the mangosteen, and (**e**) peel) (**A**), and the value added from mangosteen pericarps to xanthones extract (**B**).

A mangosteen contains 83% pericarps, 15% pulp, and 2% seeds by weight [4]. Mangosteen pericarps are a by-product of mangosteen processing (Figure 1A). Mangosteen pericarps are a nutrient-rich by-product which contains 82.50% carbohydrate, 6.45% fat, 3.02% protein, 2.17% ash, and 2.17% free sugars [5]. Mangosteen pericarps contain several health-beneficial and functional phenolic compounds, including xanthones, benzophenones, tannins, flavonoids, and anthocyanins [6]. Mainly, xanthones are abundantly found in the mangosteen pericarp compared to the other phenolics. It is estimated that there are 200 different types of xanthones are present in nature, with the majority belonging to the families of Bonnetiaceae, Clusiaceae, and Podostemaceae. There are more than 40 xanthones in *Mesua thwaitesii*, especially mangosteen [7]. The xanthone compound is a powerful bioactive compound that exhibits excellent health benefits. It acts as an antioxidant, antimicrobial, anti-cancer and anti-tumor molecule and has high resistance against the virus [8]. Typically, the fresh mangosteen pericarp has a low economic value (0.2 USD per 1 kg). However, it contains many beneficial properties and can be converted to a valued added product when the functional chemicals, particularly xanthones from the pericarp, are extracted. Studies have proven that 1 kg of fresh mangosteen could provide 2 g of pure xanthones, which is 15 times higher than the cost of fresh pericarp alone [9] (Figure 1B). Therefore, the extraction process of pure xanthones plays a significant function in many industries. There are several methods of extraction and purification of xanthones from mangosteen pericarps, and the extracts obtained can be commercialized. Therefore, the collection of knowledge about the extraction techniques and various biological effects of xanthones from mangosteen pericarp are extensively discussed in this review.

## 2. Characteristics of Mangosteen

**Figure 2:**
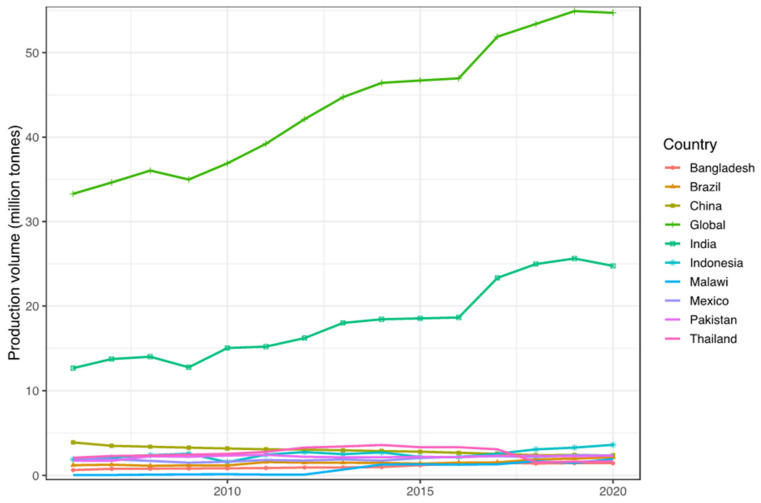
The top 10 producers of fresh mangosteen in the world. Data [11].

The mangosteen plant is native to Indonesia and Malaysia. The most cultivated mangosteen is *G. mangostana var. mangostana* and another two varieties of mangosteen are wild species, *G. mangostana var. malaccensis* and *G. mangostana var. borneensis* [10]. It is cultivated on a large scale in countries such as Bangladesh, Brazil, China, India, Indonesia, Malaysia, Malawi, Mexico, Pakistan, and Thailand (Figure 2), and for small-scale production in other countries, including Australia (Southern Queensland, New South Wales), Cambodia, Cuba, Dominica, Ecuador, Guatemala, Honduras, Jamaica, Laos PDR, Madagascar, Myanmar, Panama, Puerto Rico, Singapore, Sri Lanka, Trinidad, Tobago, USA (Florida, Hawaii), and Vietnam. Mangosteen is a tropical evergreen tree categorized as slow-growing, erect with a pyramidal crown, and normally the plant height ranges between 6 and 25 m.

The mangosteen tree has dark brown or nearly black flaking bark, and the tree’s inner bark contains yellow, gummy, and bitter latex [12]. At the same time, the leaves are short-stalked, ovate-oblong, or elliptic, leathery, thick, dark green, slightly glossy above, yellowish-green, and dull beneath. The leaf size is around 9 to 25 cm long and 4.5 to 10 cm wide, with a conspicuous, pale midrib [13]. Flowers are 4–5 cm wide, fleshy, male, or hermaphrodite on the same tree, with four sepals and four ovate. The fruit is rounded, dark purple to red–purple, and smooth externally, 3.4–7.5 cm in diameter and weighs 70–150 g; the rind is 6–10 mm thick, with four to eight triangular segments of snow-white, juicy, soft flesh [14]. Mangosteen trees require an average annual rainfall of around 1270 mm, the ideal temperature for mangosteen growth is between 25 and 35 °C, with more than 80% relative humidity, and this tree can grow well in coarse soils, slit and is able to be well-drained. Mangosteen can be cultivated in a wide range of altitudes, between 76 and 500 m above sea level [15].

## 3. Xanthones in the Mangosteen Pericarp Uses

### 3.1. Xanthones in the Mangosteen Pericarp

**Figure 3:**
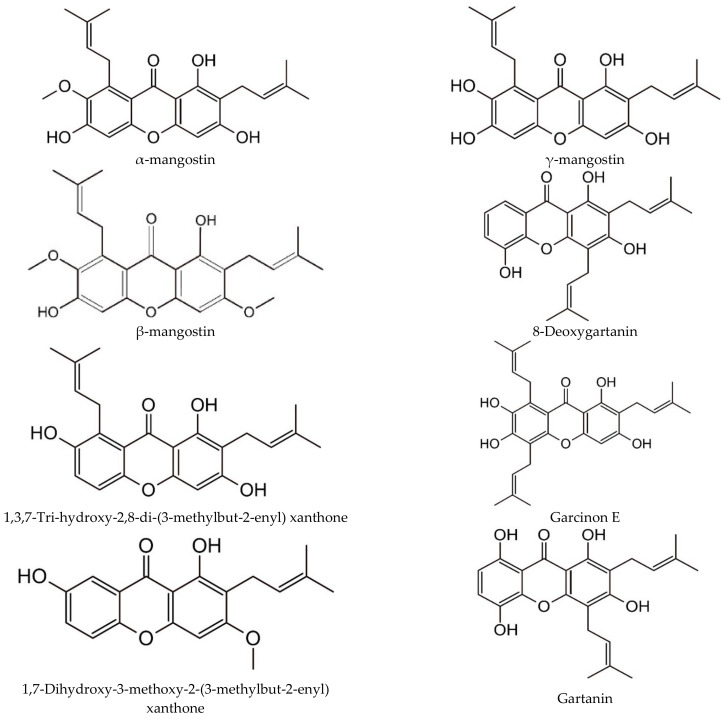
Chemical structure of major and minor xanthone compounds.

Xanthones (CH_3_H_8_O_2_) are found in various parts of mangosteen, such as heartwood, bark, yellow gum (or yellow latex), leaves and pericarp (Figure 3). Studies have found that the concentrations of α-mangostin and γ-mangostin in the yellow gum collected from the outside of mangosteen pericarp were 382.2 and 144.9 mg/g wet basis, respectively [11]. The concentrations of α-mangostin and γ-mangostin in yellow gum were approximately six times and seven times higher than those in the mangosteen pericarp. The yellow gum is a rich source of xanthones in mangosteen fruit, followed by the pericarp and pulp [11]. The main components in the yellow gum of mangosteen are triterpenoids, flavonoids, and tannins, while mangosteen pericarps contain lignin, phenolic, benzophenones and xanthones and these compounds exhibit a strong pharmacological effect [16]. α-mangostin is the most important xanthone found at 69.02% in the mangosteen pericarp, followed by γ-mangostin, which is around 17.86%. Additionally, the minor compounds (13.13%), including gartanin, 8-deoxygartanin, garcinon E, 1,7-dihydroxy-3-methoxy-2-(3-methylbut-2-enyl) xanthone, and 1,3,7-tri-hydroxy-2,8-di (3-methylbut-2-enyl) xanthone, are also found in the pericarps. Furthermore, anthocyanin pigments (31.29%) and tannin (7.14%) are also found in the mangosteen pericarp [7]. The pericarp of mangosteen contains ten times higher levels of phenolics than the pulp; furthermore, pericarp antioxidant activity is twenty times higher than in the pulp. The extraction of mangosteen pericarp by organic solvents leads to the separation of polar and non-polar compounds. Non-polar components include xanthones and their prenylated benzophenone, and polar compounds consist of catechins, procyanidins, and anthocyanidins. Ethanol has been used to extract xanthones from mangosteen pericarp.

According to Rizaldy et al. [17], 14 types of xanthone derivatives were found in mangosteen pericarp, which include 7-odemethylmangostanin, mangostanin, 8-deoxygartanin, gartanin, garcinone E, trapezifolixanthone, padiaxanthone, tovophyllin A, 1,5,8-trihydroxy-3-methoxy-2 [3-methyl-2-butenyl] xanthone, garcinone B, 1,3,7-trihydroxy-2,8-di-(3-methylbut-2-enyl) xanthone, mangostenone D, mangostinone, and 1,7-dihydroxy-2-(3-methylbut-2-enyl)-3-methoxyxanthone.

### 3.2. Bioactivity of Xanthones

#### 3.2.1. Experimental Studies

The α-mangostin from mangosteen pericarp is the major component of xanthones. Many biological activities and pharmacological properties are associated with it, including antioxidant activity, antimicrobial properties, anti-obesity properties, anti-inflammatory properties, anti-hyperglycemia properties, anti-diabetic properties, anti-neoplastic properties, anti-proliferative properties, apoptosis-inducing properties and anti-tumor properties. The γ -mangostin is the second most common compound in mangosteen pericarps. It inhibits the digestion of carbohydrates with the action of the α-glucosidase enzyme. It is in the small intestinal wall and used to digest the starch and carbohydrates into single sugar molecules through enzymatic hydrolysis. It impairs glucose absorption into the bloodstream and helps reduce sugar levels in the blood. In addition, γ-mangostin has also exhibited antioxidant activity, anti-inflammatory, anti-cancer, and neuroprotective effects. β-mangostin can hinder the increase of glioma cells, the most public and assertive virulent nervous system tumor. Two other bioactive xanthones that appear in mangosteen pericarps are garcinone E and gartinin. The garcinone E inhibits ovarian cancer cells and has an anti-proliferative effect on cancer cells including cervical, liver, stomach, breast, and large intestinal cancers. Gartinin has a preventive effect on cervical cancer cells and lymphoma [18]. The important health properties of pure xanthones extracted from mangosteen pericarp include anti-acne [19], anti-inflammatory, anti-irritating, antibacterial, and anti-blackmark effects [20], protection against free radicals [21], and skin whitening [22]. Currently, mangosteen pericarp extract and mangosteen xanthones are widely used in the medical, food supplement, oral care, and cosmetic industries. In the medical industry, pericarp extract from mangosteen is used as a raw material to produce various oral-based health products, including topical anti-inflammatories to reduce itching caused by fungi and medicinal mixtures to treat various ulcers. In the supplementary food industry, mangosteen pericarps are used to develop various dietary supplements, such as antioxidant and anti-ageing supplements, to enhance immunity. In the oral care industry, the extract from mangosteen pericarp is developed into various oral-based hygienic products such as mouthwash, toothpaste, a mixture of dental floss, and spray to remove bad breath. In the food industry, mangosteen pericarp extract is used to develop into products for consumer consumption, such as antioxidant snack-based foods, foods to increase energy, snack bars, rice powder, and healthy drinks [23]. In the cosmetic industry, pure xanthones from mangosteen pericarp are used to develop various skin care products designed to control acne and ageing, and some xanthones products promote good skin health and increase skin brightness [24].

A study from Narasimhan et al. [25] found that xanthones extracted from mangosteens inhibit the tyrosinase activities in the skin and, consequently, control the production and accumulation of melanin. γ-mangostin exhibited an anti-hyaluronidase activity and protected the skin from hyperpigmentation [21]. Janardhanan et al. [24] found that the pericarp extract of mangosteen inhibited several cariogenic bacteria, such as *Streptococcus* mutants, *S. sanguis, S. salivarius, S. oralis* and *Lactobacillus acidophilus* [24]. An α-mangostin is the main component from the pericarp extract that demonstrated antimicrobial activities, mainly antibacterial, against *Escherichia coli*, *Staphylococcus aureus, Bacillus subtilis*, and *Pseudomonas aeruginosa,* as well as anti-fungal effects against *Aspergillus niger* and *Candida albicans* [25,26]. Furthermore, Leelapornpisid [26] found that the pericarp extract from the mangosteen exhibited anti-plaque properties and controlled severe dental cavities. The in vitro study from Koh et al. [27] found that the application of xanthones from mangosteen pericarp exhibited significant inhibitory activity against the *Mycobacterium tuberculosis,* which was done by disrupting the inner membrane and led to ATP depletion with no cross-resistance with the current anti-TB drugs.

#### 3.2.2. Molecular Docking Studies

Using the molecular docking technique to model the interaction between a small molecule and a protein at the atomic level [28], we can define the behavior of small molecules at the binding sites of target proteins and understand basic biochemical processes. Many molecular docking studies define the biological activities of xanthones inappropriately [20,29,30,31,32]. Mangostins and garcinones from mangosteen pericarps were found to have anti-ageing properties against enzymes such as matrix metalloproteinase 1 (MMP1), nuclear export protein (NEP), and prophenoloxidase (PPO3) [20]. Furthermore, the MMPs also damage the blood–brain barrier, consequently inducing hemorrhage, neuronal inflammation, and neuronal cell death [29]. A study from Siahaan et al. indicated that mangosteen extract is a potential drug for treating traumatic brain injury (TBI) by inhibiting the expression of MMP-2 and MMP-9 in the animal model [30].

The anti-diabetic drugs α-mangostin and γ-mangostin have been shown to control PPAR- γ receptors, the diphenyl peptidase-4 (DPP-4) enzyme, and aldose reductase [31]. The binding affinity showed more negative or almost similar affinity binding values than the several ligand-based drugs on the market. It also demonstrated several models of protein-bound bonds, including hydrogen bonding, allowing these compounds to be predicted in anti-diabetes drugs such as thiazolinediones (TZDs), which target PPAR- receptors [31]. In the study of 272 xanthones against seven fungal and two viral enzymes, they were reported as possible antimicrobial and antiviral agents. [32]. Miladiyah et al. demonstrated that xanthone derivatives could be effective as potential COX-2 inhibitors, which indicated the possibility of anti-inflammation activity [33].

### 3.3. Uses of Xanthones

Currently, the crude and pure xanthone extract from the pericarp of mangosteen is used widely in several industries, including medical, food supplement, oral care, food, and cosmetic industries [23]. Xanthones can be used as an oral product in the medical industry to cure ulcers and as a topical ailment to control itching and any inflammatory problems [23]. Mangosteen pericarp extract has been used to develop various dietary supplements in the food industry, enhancing immunity and antioxidant activities and aiding early ageing problems [23]. Extracts from mangosteen pericarp are used to produce mouthwash, toothpaste, dental floss, and mouth spray are a few significant products that are widely developed in the oral care industries [23]. Several products can be developed with mangosteen pericarp extracts in the food industry, such as antioxidant snack products, snack bars, and healthy beverages [23]. In addition, pure xanthone extracts from mangosteen pericarps can also be used to produce various supplementary and pharmaceutical-based products. Mangosteen pericarp extracted using ethyl acetate exhibits an inhibitory effect against the tyrosinase enzyme, which play a major role in melanin synthesis [34]. Although the pericarp extract from mangosteen showed potent antioxidant, anti-collagenase and anti-elastase activities, while γ-mangostin showed potent anti-hyaluronidase [20], the crude extract of mangosteen is a dark brown to black color, and when using this extract as an ingredient in high-value cosmetic product formulations (facial care products), it will cause the products to have an appearance that is not friendly to consumers and will make the products look inferior. Furthermore, the crude extract also retains a very small amount of xanthones [23]. Therefore, the extraction process of pure xanthones plays an important role in many industries producing value-added products from mangosteen. The bioactivities of xanthones and common uses of mangosteen pericarps extract are summarized in Table 1.

## 4. Extraction of Xanthones from Mangosteen Pericarps

### 4.1. Mangosteen Pericarp Preparation

Generally, the mangosteen fruit pericarp for xanthone extractions is selected based on the fruit maturity and ripening conditions. Studies have reported that the mangosteen fruit’s maturation period is between 1 to 4 months after flowering; however, the phytochemical contents in the fruit’s pericarp vary significantly between stages of maturation [48]. Within the maturation period, the fruit will have six stages of ripening and color, and the level of sap on the pericarp acts as a key maturity index (Table 2). However, the selection of fruit pericarp can be made from an unripe greenish yellow to fully ripe purple-black color, and fruits’ pericarps, when fully grown, despite the color of the pericarp, normally have the richest level of xanthones [49]. The extraction of xanthones from mangosteen pericarp is highly possible in fresh and/or dried forms. However, fresh mangosteen pericarps have a high moisture content, which may interfere with some solvents in the extraction [50]. Furthermore, fresh fruits have a high possibility of microbial contamination, which causes the extract to spoil easily.

Therefore, several studies used dried mangosteen pericarps as a suitable raw material for xanthones extraction and are more popular than fresh mangosteen pericarps [50]. The process for the preparation of dried mangosteen pericarps powder has several steps, including grading, washing, baking, or drying, grinding into powder, packing, and storing [51]. Normally, the pericarp of mangosteen is cut into small pieces and dried by either sun-drying or oven-drying between 60 to 90 °C; the pericarps are dried to obtain 14 % moisture, which is suitable for xanthone extractions [52]. The dried pericarps are ground into powder by either stone mortars or grinders to reduce the particle size and increase the contact surface area between the raw material and the solvent. After the grinding process, the ground pericarps are passed through a sieve to make the uniformly fine powder. The powder is then packed in airtight containers and stored in dry and cool conditions to prevent pest and microbial infestations. Suvarnakuta et al. [52] found that mangosteen pericarps extracted using low-pressure superheated steam drying at 75 °C retained more xanthones in the mangosteen pericarp as compared with traditional methods. In another study, Sotong and Ruaypom [53] used a fluidized bed technique with a recirculation air temperature of around 60–80 °C to dry the mangosteen’s pericarp of mangosteen. Their study found that an increased temperature to 80 °C rapidly decreased the pericarp moisture to 13.58% without severely affecting the xanthones.

### 4.2. Extraction

Solvent extraction is a common procedure used to separate desired components from raw materials without dissolving other components in the extraction. Generally, there are two major types of extraction categories: (1) liquid–liquid extraction is used to extract a liquid from a liquid and (2) solid–liquid extraction, or leaching, is used to dissolve the desired substance out of a solid mixture [54]. Important factors affecting solid–liquid extraction are solvent properties and solid preparation properties, such as the size, surface area, encapsulation (cell wall), time, and extraction temperature [54]. The increasing solubility and diffusion rate can vary with the influence of temperature, but the decreasing viscosity of the liquid is not affected by the temperatures. However, the high temperature will cause the impurity to dissolve and may cause the desired biological substance to be damaged. The extraction of active substances in mangosteen pericarp mostly uses solid–liquid extraction [54]. The mechanism that occurs during the extraction of xanthones from solid mangosteen pericarp powder with liquid solvent can be described as follows: (a) mass transfer of solvent to the surface of mangosteen pericarp powder, (b) the surface of the raw material is coated with a solvent, (c) extraction solvent is diffused from the surface to the porosity of the raw material, (d) solute in the raw material is dissolved in the solvent and (e) the desired solute is dispersed onto the surface of the raw material [54]. A study by Kusmayadi et al. [55] found that xanthones in mangosteen pericarp were extracted with seven different major organic solvents, including ethanol, acetone, ethyl acetate, methanol, hexane, acetic acid, and water, and their study showed that all solvents had significantly differed among each other with the extracted xanthones. Among the solvents, ethanol performed best in extracting xanthones and antioxidants. In addition, their study also found that extraction time plays a key role in retaining xanthones, and when xanthones were extracted for 48 h, they were extracted at a high level using acetone, and in short-range (24 h) extraction durations, ethanol performed well in extracting xanthones. Generally, the extraction methods of xanthones can be divided into two major types, which are (1) traditional extractions and (2) modern extractions.

#### 4.2.1. Traditional Extraction

Traditional extraction is the easiest because it uses conventional solvents and heaters. This is still limited in many aspects; particularly, a large amount of solvent is required and takes a long time to extract while the productivity is low. The most used method is maceration. A study from Pojanaukij and Kajorncheappunngam [56] examined the capacity of the maceration method on xanthone extraction from mangosteen pericarp, and their study found that when extracting 1 kg of mangosteen pericarp that was macerated with 4 L of 95% ethanol for seven days, it was possible to obtain 1.19 mg/g of xanthones from the mangosteen pericarp. Additionally, dried mangosteen powder was also recommended for maceration extraction. A study from Yoswathana et al. [57] found that 5 g of dried mangosteen pericarp powder was extracted using 100 mL of 95% ethanol solvent without a shaker generating a yield of 31.55 mg/g of xanthone. Solvent extraction of mangosteen pericarp for the isolation of α-mangostin is performed directly or indirectly using solvent partitioning. The former approach involves direct contact of the plant material in a single solvent, e.g., chloroform [58] or methanol [59] or sequentially in multiple solvents [60], whereas the latter typically involves indirect extraction via successive solvent partitioning. A comparison of three different extraction methods using methanol and ethyl acetate as solvents was conducted to compare the yield of α-mangostine. This involved direct extraction with methanol and ethyl acetate and indirect extraction using solvent partitioning with an initial methanol extract. The results showed that direct extraction yields higher α-mangostine than indirect extraction (direct methanol extraction: 318 mg; ethyl acetate direct extraction: 305 mg; ethyl acetate indirect extraction: 209 mg per 5 g total dried pericarp) [61]. In another study, Kusmayadi et al. [55] studied the effects of different solvents (ethanol, acetone, ethyl acetate, methanol, hexane, acetic acid, and aquadest) and extraction times (24, 36, and 48 h) on total xanthone and antioxidant yield of mangosteen peel extract. Their result reported that the solvents and extraction time significantly affected total xanthone and antioxidant yield. Mangosteen peel extracted by acetone shows the best results on total xanthone at 48 h. Meanwhile, mangosteen peel extracted by ethanol for 24 h was the best result on antioxidant yield. 

In addition, percolation is a continuous extraction method that uses an instrument called a percolator for the xanthone extractions. A total of 10 g of powdered dried mangosteen fruit rind was combined with 10 mL of 95% ethanol, and the combination was let to stand for 1 h. A percolator was used to add 95% ethanol to the mixture once it had been moved there (3.6L). Once the percolate was gone, the extraction was carried out at room temperature with a flow rate of 3 mL/min (20h). The α-mangostin content in the extract by percolation was 12.71 % *w*/*w* [19]. Soxhlet extraction is another traditional method for determining lipid content in food samples or essential oil content in raw materials by continuous extraction using a low-boiling solvent. Soxhlet extraction was employed to extract xanthones from dried mangosteen pericarp. The results presented that the xanthones extracted by Soxhlet were 31.26 mg/g of dried mangosteen pericarp [57]. Mangosteen pericarp was dried at a temperature of 65 °C to 15% moisture content (dry basis) and then extraction with ethanol by Soxhlet extraction obtained α-mangostin content at a level of 34.82 ± 0.17 *w*/*w* from the crude extract. The main disadvantage of the Soxhlet extraction method is that it requires large solvent volumes and takes a long extraction time at high temperature that causes the degradation of heat-sensitive compounds [62]. Ethanol extracts important substances from dried mangosteen fruit pericarps with different methods, namely maceration, percolation, Soxhlet extraction, ultrasonic extraction, and extraction using a magnetic stirrer; these substances had an anti-acne effect. With Soxhlet, crude extract (26.60% dry weight), α-mangostin (13.51%, *w*/*w* of crude extract) and anti-acne activity were obtained at a concentration of 50% ethanol, an optimal amount for extraction by Soxhlet [19]. 

Infusion is an extraction over a short period using hot or cold water. A ripe mangosteen with a blackish-purple tint was used. The mangosteen pericarps were shelled and briefly wetted to preserve the secondary metabolites. Then, 1000 mL of drinking water and 330 g of pericarps were combined, and the mixture was submerged for 12 h until the water turned dark red and smelled fresh. The stock solution, which contained 1.87% of the flavonoid in the mangosteen pericarps infusion, was then obtained. Furthermore, concentrations of 0.25, 0.5, 1 and 2% were used to create the working solution. The infusion of mangosteen pericarps was prepared once every two weeks, kept in bottles covered in aluminum foil to block off light, and kept at 4 °C. Additionally, a 2% concentration of mangosteen pericarps infusion was made up [63]. The conventional methods, such as maceration, Soxhlet extraction and heat reflux, are time-consuming, high in operating costs and low in product recovery [64]. A comparison of xanthones and α-mangostin content from the mangosteen pericarp extract using traditional extraction methods is shown in Table 3.

#### 4.2.2. Modern Extraction Techniques

**Figure 4:**
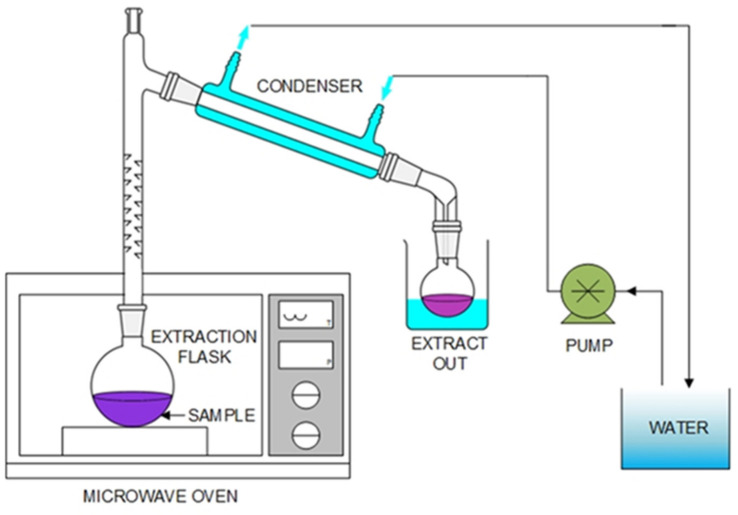
Schematic diagram of microwave-assisted extraction apparatus.

Higher yields, faster extraction times, and lower solvent contents characterize modern extraction techniques. Modern methods are implemented on an industrial scale; microwave extraction is an extraction method using microwaves, which are electromagnetic waves [66,67]. This wave is converted to heat by causing polarized particles or molecules to friction and heated up. In other words, this happens when the extracted substance is placed in an electromagnetic field. The polarity of molecules within the substance to be extracted causes resistance to movement or friction with each other, causing heat that affects plant cells and results in the extraction of active substances [68]. The principle of extraction by using microwaves is that it uses frequencies in the range of 3 × 10^2^ to 3 × 10^5^ MHz and wavelengths in the range of 0.01 to 1 m together with organic solvents to extract important substances [69]. When the extracted substance is placed in an electromagnetic field, with the polarity properties of the molecules within the extracted substance, there will be resistance to motion, causing heat, which affects the cell tissue of the extract and influences the solubility of the desired active substance. Electricity or microwave heating is caused by energy transfer from two mechanisms, dipole rotation and ionic conduction through dipole changes. This replaces charged ions in a substance and a solvent where both processes occur simultaneously. Ion transport induced by changing electric fields is called ionic conduction. If a solvent resists the movement of the ions, it results in friction and heat [70]. Modifying the dipole of a molecule in conjunction with a change in the electric field is called a dipole rotation, where the frequency of 2450 MHz causes microwaves to change the electric component at a speed of 4.9 × 10^4^ times per second, thus generating frictional heat [71]. Energy transfer is a key feature of microwave heating; 2450 MHz is the most popular frequency, with a power range of 600–700 watts [72]. Usually, the heat transfer of traditional extraction processes is the energy transferred to the raw material to be extracted by convection, conducting, and radiation. In the case of microwave extraction, the microwaves are converted to heat by causing polarized particles or molecules to cause friction and generate heat. In microwave extraction, the effectiveness of the extraction depends on the use of suitable conditions (Figure 4).

The factors affecting microwave extraction include the solvent system and solvent-to-powder ratio (solvent nature and solvent feed ratio; S/F ratio), microwave power, extraction temperature, extraction time and cycle, plant matrix characteristics, and the effect of stirring [66]. For example, the microwave-assisted extraction (MAE) of xanthones from mangosteen pericarps showed that the optimum conditions for the extraction of antioxidant-rich xanthones are the 2.24 min irradiation time, the solvent ratio of mangosteen pericarps powder of 25 mL/g and a 71% ethanol concentration [22]. A study from Ghasemzadeh et al. [73] found that mangosteen pericarp was extracted using 72.40 % (v/v) ethyl acetate with microwave extraction, and the conditions were set to 189.20 W output power and 3.16 min irradiation time, which was able to extract xanthones, particularly α-mangostin, at about 120.68 mg/g of mangosteen pericarp (dry matter). Microwave-assisted extraction has been successfully applied to extract bioactive compounds from plant matrices, which needed hours to complete with conventional methods. There are many advantages of MAE, including reduced extraction time, reduced solvent usage, improved extraction yield and environmental friendliness [64].

**Figure 5:**
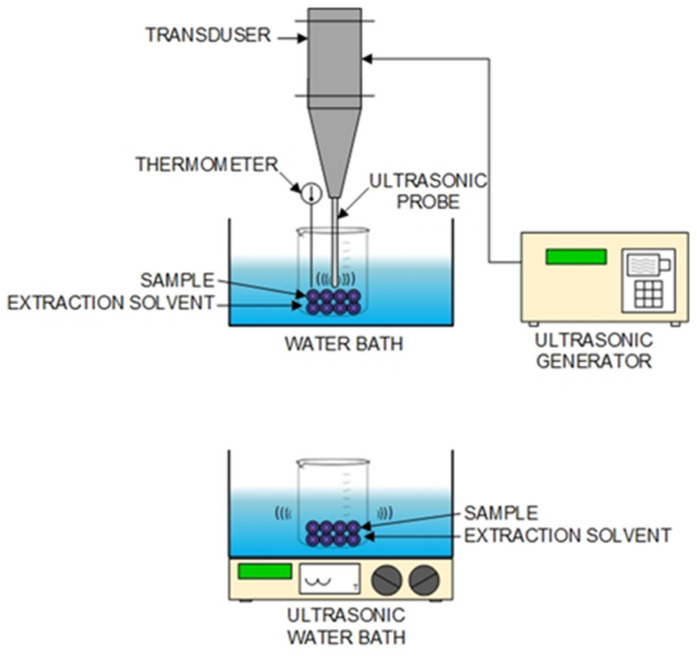
Schematic diagram of ultrasonic-assisted extraction apparatus.

In ultrasonic extraction, the resulting supersonic energy has chemical, biological, and physical effects through a phenomenon known as cavitation, which is a phenomenon that occurs when many small bubbles exist in the liquid body, the surface of the solid, or the surface of the container containing the liquid where compression and amplification due to the pressure of supersonic waves occur. This causes the bubbles to grow and shrink as the gas inside the liquid diffuses in and out of the bubbles alternately, causing a strong stirring effect. This can increase the efficiency of solid–liquid extraction (Figure 5).

Gases and solid particles in the extraction medium are the major factors influencing cavitation. In addition, the pressure inside the liquid, the viscosity of the liquid, the frequency of sound waves, the temperature, and the intensity of the supersonic waves could also affect the cavitation. For example, a comparative study from Yoswathana [65] using three different extraction methods (ultrasonic-assisted extraction, Soxhlet extraction, and maceration) to extract xanthones from mangosteen pericarp found that the optimum conditions for extracting xanthones from mangosteen pericarp were a temperature of 33 °C, an amplitude of 75, and 80% ethanol; the ultrasonic-assisted extraction method for 0.5 h, Soxhlet extraction at 2 h, and maceration at 2 h could able to extract xanthones at a concentration of 0.1760, 0.1221, and 0.0565 mg/g of dry mangosteen, respectively. Ultrasonic extraction is proven to be an economical and effective method. It is an alternative technique at the laboratory or industry scale due to shorter extraction time and higher extraction yield [74]. Ultrasonics can penetrate the cellular wall, reduce the particle size, and increase the mass transfer between the cell walls and the outside because of the cavitation effect [75]. Fresh mangosteen rind was mixed with 95% (v/v) ethanol and extracted in an ultrasonic bath, which generated the frequency of 30 kHz, for 10 min at room temperature, which was able to yield the α-mangostin and 8-desoxygartanin contents at a level of 47.82 ± 3.76 mg/g and 1.43 ± 0.30 mg/g (d.b.), respectively [52].

**Figure 6:**
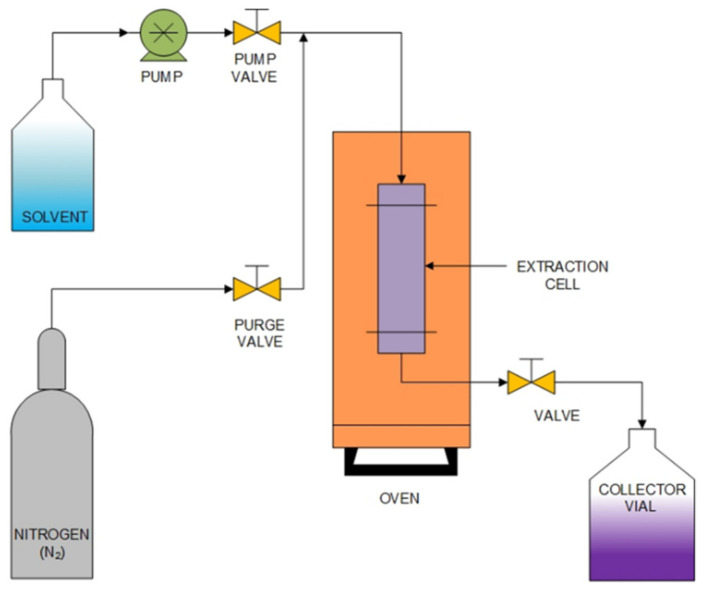
Schematic diagram of pressurized liquid extraction apparatus.

Pressurized liquid extraction (PLE) is a technique that involves extraction using liquid solvents at elevated temperature and pressure, which enhances the extraction performance as compared to those techniques carried out at near room temperature and atmospheric pressure [76,77]. The enhanced solubility and mass transfer properties are the merits of enabling the use of solvents at temperatures above their atmospheric boiling point. This technique is also known as accelerated solvent extraction, pressurized liquid extraction, and enhanced solvent extraction. In the case when water is used as the extraction solvent, the technique is referred to as pressurized hot water extraction, sub-critical water extraction or superheated water extraction. Subcritical water extraction of phenolic compounds from mangosteen pericarps was examined at temperatures of 120–180 °C and pressures of 1–5 MPa. In a batch system, the maximum yield of xanthone was 34 mg/g sample at 180 °C and 3 MPa with 150 min reaction time [78]. The development of a subcritical water extraction method using deionized water, which contains a deep eutectic solvent at 10–30% volume, was used as an extraction medium instead of water. The xanthone and phenolic compounds extracted in 10% deep eutectic solvents at 160 °C, and 5 MPa for 180 min in the semi-batch system were yielded at a level of 27.15 mg/g dried sample and 372.69 mg of GAE/g dried sample, respectively. Adding deep eutectic solvent in the subcritical water extraction process could accelerate the hydrolysis reaction to extract the plant biomass components matrix [79]. In addition, the use of natural deep eutectic solvents (NADES) as a green solvent for the extraction of α-mangostin from the pericarp of mangosteen was studied [80]. The NADES consisted of choline chloride, a quaternary ammonium salt, and four hydrogen bond donors: 1,2-propanediol, citric acid, glycerol, and glucose. The highest α-mangostin extraction yield of 2.6 % (*w*/*w*) was garnered from dried pericarp using a mixture of choline chloride and 1,2-propanediol in a 1:3 mole ratio. The results conclude that NADES made of choline chloride and diol-based hydrogen bond donors are adequate for extracting bioactive compounds from the mangosteen pericarp. This method is widely used because of its good extraction efficiency compared to other methods and as a clean technology because it does not use organic solvents. This new method uses a liquid solvent under pressure (Figure 6).

**Figure 7:**
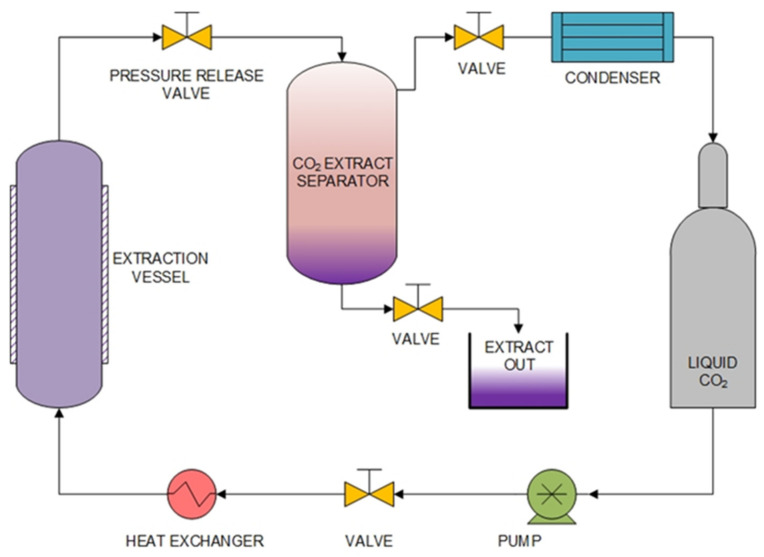
Schematic diagram of supercritical fluid extraction apparatus.

Carbon dioxide is determined to be in the superfluid or supercritical SC-CO_2_ (T_c_ = 31.2 °C and P_c_ = 7.28 MPa) with the properties of CO_2_ compressed to a water-like liquid and being flow-like (Figure 7) [81]. Supercritical fluid extraction has received much attention, especially in the food and pharmaceuticals industry, because it presents an environmentally responsible and efficient extraction technique for solid materials that were introduced and extensively studied for the separation of active compounds from herbs and other plants [82]. Normally, the air is allowed penetrate the material to be extracted. This method is generally used to extract low-polarity substances. However, it has now been applied to allow the extraction of more polar substances by adding a more polarized organic solvent to the extraction, known as a co-solvent [83]. Extraction of α-mangostin from mangosteen pericarps by supercritical fluid extraction (SFE) techniques using CO_2_ as a solvent under optimal operating conditions at a temperature of 40 °C and a pressure of 10 MPa, the mole fraction of extracted α-mangostin reached 4.5 × 10^−7^ [84]. Furthermore, the improved supercritical fluid extraction by ethanol-modified SC-CO_2_ (SC-CO_2_ + 4% ethanol) extraction performed at 20 MPa and 40 °C provides good antioxidant activity and is a suitable technique for the concentration of bioactive compounds from mangosteen [85].

Compared with the PLE technique, the SFE technique can extract exceptionally high levels of xanthones with good purity. A comparison of xanthones and α-mangostin content from the mangosteen pericarp extract using modern extraction methods is shown in Table 4.

A review of modern extraction techniques reveals that the microwave-assisted extraction method has many advantages, such as low solvent consumption, less time consumption, and fast energy transfer through the irradiation that permits the good diffusion of solvent within the extraction medium, especially for the preparation of antioxidant-rich plant extracts. M’hiri et al. [86] have evaluated that microwave-assisted and ultrasound-assisted extraction could extract the highest value of total phenolic content and antioxidant activity of the plant. In addition, microwave-assisted extraction methods have been studied for scaling up to an industrial scale [87]. Thus, has MAE led to the most prevailing extraction techniques compared to other methods: conventional solvent extraction, ultrasonic-assisted extraction, high-pressure extraction, and supercritical fluid extraction. In terms of stability, mangosteen pericarp extract has been developed into a throat spray, and research has been conducted on the stability of the extract by using the mangosteen pericarps throat spray, which contains 1% of the crude mangosteen extract, by checking its stability at 4 °C, 30 °C, 40 °C and room temperature for 180 days. The throat spray samples were found to be quite stable for up to 180 days at all tested conditions [88].

## 5. Conclusions

Mangosteen pericarp is a potential source of natural xanthones and can be used as raw materials for xanthone compound extraction to apply in the healthcare industry. The information from this preliminary gathering may benefit those interested in the benefits of mangosteen pericarp and xanthones extraction methods. Data from various extraction methods show that modern extraction methods provide higher quantitative and temporal efficiency than traditional extraction methods. In the future, extraction methods may combine several methods or develop new techniques for better efficiency. This also includes the creation of guidelines for their further use in other industries. Building knowledge on developing suitable machinery and technology for industrial xanthone extraction processes may support xanthone extraction’s quantity and quality. Methods for extracting xanthones from mangosteen pericarps must be developed in quantity and quality, including scaling up to industrial production. The extraction method must be environmentally friendly for sustainability. Moreover, there must be research on developing foods, cosmetics, and medical products. Future studies and developments on how to use xanthones in various aspects are recommended to expand the utilization of industrial by-products or local fruit wastes in accordance with the Bio-Circular-Green Economy approach (BCG). This refers to the cost-effective use of biological resources while maintaining environmental balance.

## Figures and Tables

**Table 1:** The bioactivities of xanthones and common uses of mangosteen pericarps extract.

Bioactivity/Use		Component	References
**Bioactivities**	antioxidant	α-mangostin	[18,19,20,21]
		γ-mangostin	[20]
		crude	[19,20,22,35]
	anti-obesity/ anti-amylase/anti-glucosidase/anti-lipase	α-mangostin	[36]
	anti-inflammatory	α-mangostin	[37,38]
		γ-mangostin	[38]
	anti-hyperglycemias, anti-diabetic	α-mangostin	[39]
		γ-mangostin	[40]
	anti-neoplastic, anti-proliferative, anti-cancer	α-mangostin	[41]
		γ-mangostin	[42]
		β-mangostin	[43]
		crude	[44]
	anti-apoptosis	α-mangostin	[35]
	neuroprotective effects/ brain protective effect	α -mangostin	[45]
		crude	[30]
	anti-tyrosinase/ anti-collagenase/anti-elastase/ anti-hyaluronidase	α-mangostin	[20,35]
	anti-bacteria	α-mangostin	[19,20,21,25,26]
		crude	[13,19,24]
	anti-TB	xanthone derivatives	[27]
	anti-fungi and yeast	α-mangostin	[13,25,26]
**Uses**	cosmetic/ anti-acne	crude	[13]
	food industry/ food supplement	crude	[46]
	oral care	crude	[47]
	deodorant	crude	[21]

**Table 2:** The mangosteen ripening in all six levels.

Level	Apparent
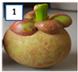	5–50 % pink dots on a greenish-yellow background. The fruit is unripe and has a lot of gum. It’s difficult to separate the mangosteen pulp from the pericarps.
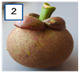	Dots spread 51–100 %, yellow-green with pink. The fruit is practically ripe, and the gum level is less than 1. It’s difficult to separate the pulp from the pericarps of mangosteen fruit.
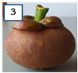	Although the spots are not as apparent as at level 2, the mangosteen pulp can be separated from the pericarps.
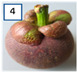	Mangosteen pulp can be removed from the pericarps and eaten, ranging in color from red to purple red.
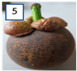	Mangosteen pulp is readily extracted from the pericarps and has a dark purple color. It is ready to eat and has no gum.
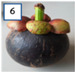	The purple and black color pericarp represent the perfectly ripe fruit, ready to eat.

**Table 3:** A comparison of xanthones, α-mangostin content from the mangosteen pericarp extract by traditional extraction.

Extraction Method	Conditions	Xanthone Compounds	Reference
Maceration	1 kg of mangosteen pericarp that macerated with 4 L of 95 % ethanol for 7 days, and was able to obtain 1.19 mg/g of xanthones from the mangosteen pericarp	Xanthones	[56]
Maceration	The optimum conditions for extracting xanthones from mangosteen pericarp were at 33 °C, amplitude was set to 75, and 80% ethanol by the maceration at 2 h could be able to extract xanthones at a concentration of 0.0565 mg/g of dry mangosteen.	Xanthones	[65]
Percolation	10 g of powdered dried mangosteen fruit rind was combined with 10 mL of 95 % ethanol, and the combination was let to stand for 1 h. A percolator was used to add 95% ethanol to the mixture once it had been moved there (3.6 L). Once the percolate was gone, the extraction was carried out at room temperature with a flow rate of 3 mL/min (20 h). The α-mangostin content in extract by percolation was 12.71 % *w*/*w*	α-mangostin	[19]
Soxhlet	Soxhlet extraction was employed to extract xanthones from dried mangosteen pericarp. The results presented that the xanthones extracted by Soxhlet, 31.26 mg/g of dried mangosteen pericarp	Xanthones	[57]
Soxhlet	crude extract (26.60 % dry weight), α-mangostin (13.51%, *w*/*w* of crude extract) and anti-acne activity, were obtained at a concentration of 50 % ethanol, an optimal amount for extraction by Soxhlet	α-mangostin	[19]
Soxhlet	The optimum conditions for extracting xanthones from mangosteen pericarp were 33 °C, amplitude was set to 75, and 80% ethanol by Soxhlet extraction at 2 h, which was able to extract xanthones at a concentration of 0.1221 mg/g of dry mangosteen.	Xanthones	[65]
Infusion	1000 mL of drinking water and 330 g of pericarps were combined, and the mixture was submerged for 12 h until the water turned dark red and smelled fresh. The stock solution, which contained 1.87 % of the flavonoid in the mangosteen pericarps infusion, was then obtained.	Flavonoid content such as xanthones, tannins, and catechins.	[64]

**Table 4:** A comparison of xanthones and α-mangostin content from the mangosteen pericarp extract by modern extraction techniques.

Extraction Method	Conditions	Xanthone Compounds	Reference
Microwave	The optimum conditions for the extraction of antioxidant-rich xanthones are the 2.24 min irradiation time, the solvent ratio of mangosteen pericarps powder of 25 mL/g and a 71% ethanol concentration.	Xanthones	[22]
Microwave	The mangosteen pericarp was extracted using 72.40 % (*v*/*v*) ethyl acetate with microwave extraction, with 189.20 W output power and irradiation time was set to 3.16 min, which was able to extract xanthones, particularly α-mangostin, at about 120.68 mg /g of mangosteen pericarp (dry matter).	α-mangostin	[73]
Ultrasonic	The optimum conditions for extracting xanthones from mangosteen pericarp were 33 °C, an amplitude of 75, and 80% ethanol by the ultrasonic-assisted extraction method for 0.5 h, which could extract xanthones at a concentration of 0.1760 mg/g of dry mangosteen.	Xanthones	[65]
Subcritical Water Extraction	Extraction of xanthone compounds from mangosteen pericarps was examined at temperatures of 120–180 °C and pressures of 1–5 Mpa using a batch extractor. The maximum yield of xanthone was 34 mg/g sample at 180 °C and 3 Mpa with 150 min reaction time.	Xanthones	[78]
The supercritical fluid extraction technique	Extraction of α-mangostin from mangosteen pericarps by supercritical fluid extraction technique using CO_2_ as a solvent under 35, 40 and 50 °C, pressure 10 to 20 Mpa could extract α-mangostin up to 4.5 × 10^−7.^	α-mangostin	[84]

## Data Availability

Not applicable.
